# Effect of Neuromuscular Training and Neurodynamic Solutions for Asymptomatic Prolapsed Intervertebral Disc and Coexisting Piriformis Syndrome in a 19-Year-Old: A Comprehensive Case Report

**DOI:** 10.7759/cureus.53050

**Published:** 2024-01-27

**Authors:** Prajyot Ankar, Grisha R Ratnani, Swapnil U Ramteke, Pratik R Jaiswal

**Affiliations:** 1 Sports Physiotherapy, Ravi Nair Physiotherapy College, Datta Meghe Institute of Higher Education and Research, Wardha, IND

**Keywords:** neuromuscular training, neurodynamic solutions, sciatica, prolapsed intervertebral disc, rehabilitation, piriformis syndrome

## Abstract

This case study examines the treatment journey of a 19-year-old male who presented with bilateral buttock pain, lower limb weakness, and instability caused by piriformis syndrome and asymptomatic Prolapsed intervertebral disc (PIVD) herniation. The intervention strategy was guided by clinical assessments, including neurological and musculoskeletal evaluations, as well as confirmatory magnetic resonance imaging (MRI) findings. The patient's treatment plan adopted a comprehensive approach that incorporated neuromuscular training and neurodynamic solutions. The former focused on strengthening the core and lower limb muscles to correct biomechanical imbalances associated with piriformis syndrome. Concurrently, neurodynamic solutions, such as targeted stretching and mobilization exercises, were employed to alleviate sciatic nerve compression related to asymptomatic PIVD. The results demonstrated significant improvement in symptoms, highlighting the effectiveness of the individualized rehabilitation program. This case report underscores the success of a multifaceted approach in addressing the intricate interaction between muscular and neural components in piriformis syndrome and asymptomatic PIVD. However, further research is necessary to validate the broader applicability of this combined therapeutic strategy.

## Introduction

Piriformis syndrome and prolapsed intervertebral disc (PIVD) are two distinct musculoskeletal conditions that can have an impact on an individual's functional abilities and quality of life. According to the joint statement of the American Society of Neuroradiology, the American Society of Spine Radiology, and the North American Spine Society, disc herniation is "localized or focal displacement of disc material beyond the limits of the intervertebral disc space". Three-quarters are degenerative cases and one-fourth of disc pathology instances are true disc herniations [1]. Lumbar nerve root mechanical compression or chemical irritation results in discomfort on one side of the body [2]. A twisting mechanism in combination with an axial load or flexion in connection with an axial force may be involved in the biomechanics of lumbar PIVD [3]. The incidence of a herniated disc is around five to twenty cases per thousand persons annually, with a female-to-male ratio of 1:2. It is commonly seen in adults in their 3rd or 5th decade of life [4]. The biology of the intervertebral disc is thought to change in a number of ways that lead to disc herniation. These include an increase in the amount of type I collagen in the inner annulus fibrosus and nucleus pulposus (NP), as well as the retention of water in these structures also the disintegration of collagen and extracellular matrix components [5]. It may happen at any spinal level but the most common lumbar spinal levels to observe it are L4-L5 and L5-S1 [6]. Radicular discomfort can result from pressure on the spinal nerve crossing the disc due to disc herniation and inflammation. The risk factors for PIVD include bad posture, extended periods of sitting, improper lifting technique, obesity, pregnancy and falls from heights [7].

Piriformis syndrome is a common cause of lower back pain and is often misdiagnosed due to its resemblance to various different illnesses. Abnormalities in the piriformis muscle like inflammation, hypertrophy, or anatomical changes, can cause the syndrome. Patients with low back discomfort have reported incidence rates of piriformis syndrome ranging from 5% to 36% [8]. Piriformis syndrome presents as a medical condition where the piriformis muscle becomes swollen, causing compression on the sciatic nerve or its various divisions, including the tibial and common fibular divisions. In specific instances, the common fibular division may pass over the piriformis muscle, resulting in increased hip pain while driving or sitting. Surgical confirmation and an intraoperative electromyography (EMG) study are utilized to diagnose this condition, with a trans-gluteal surgical approach proving successful in resolving the issue. It is estimated that approximately 16% of individuals experience this condition, making it the most prevalent form of extraspinal sciatica [9-10].

A growing number of research suggests that neural treatment might speed up sciatica patient's rehabilitation. The main aim of neural management is to mobilize surrounding tissues and nerves in order to restore peripheral nervous system balance. The sciatic nerve's neurodynamics may be restored with the help of neural treatment. Changes in nerve function can be linked to the sciatic nerve compression. Furthermore, the restoration of nerve mobility is related to the amelioration of clinical symptoms in individuals with sciatica [11]. Neurodynamics is a field that combines the mechanics and physiological functions of the nervous system, allowing for the evaluation and treatment of patients through neural mobilization and manual therapy. It covers the interactions between mechanics and physiologic processes, while pathodynamics refers to the combination of pathophysiological and pathomechanical events in disorders. Physical therapists aim to improve pathodynamic changes in patients with pain syndromes, reducing symptoms and disability [12].

## Case presentation

Patient information

**Figure 1:**
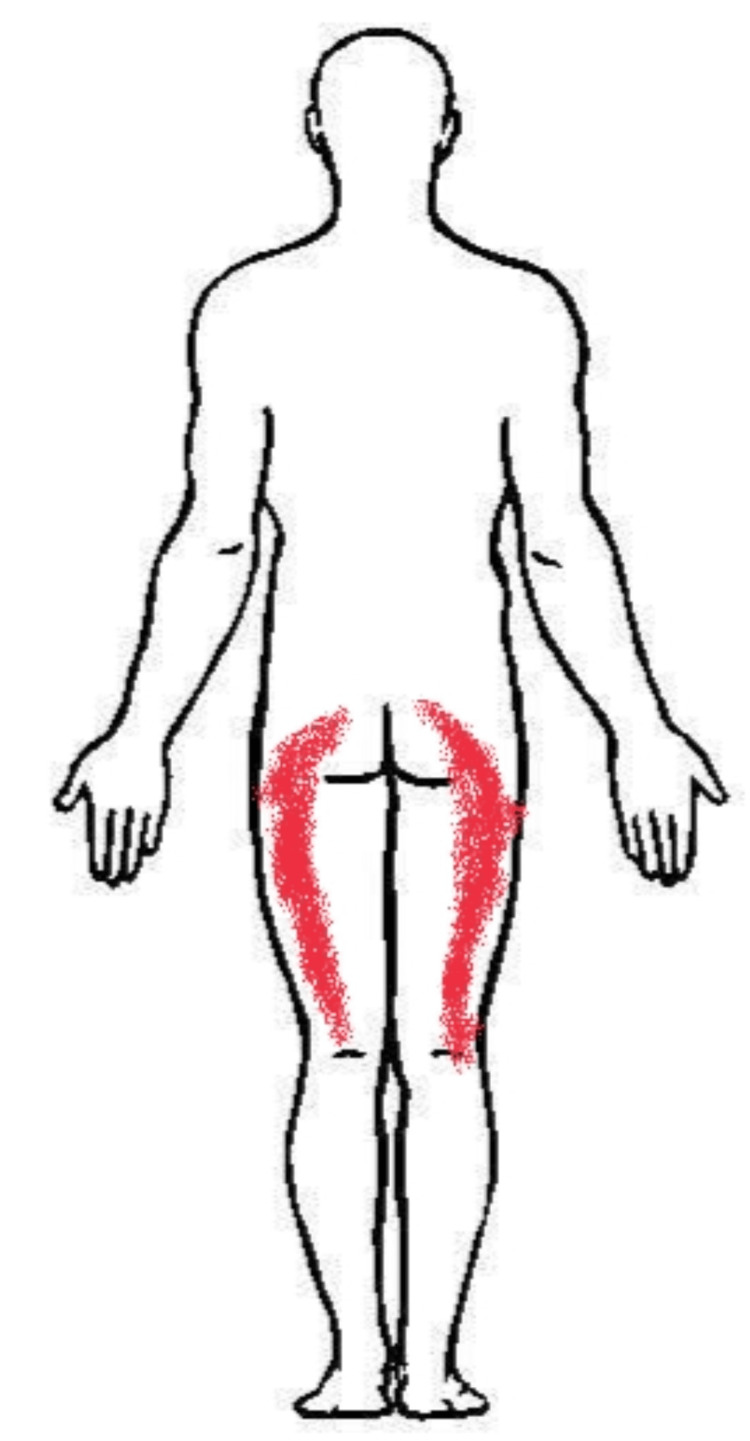
Body chart of reported symptoms. The red zone area represents the area of pain. Image credits: Prajyot Ankar

This report represents a case of a 19-year-old male who was a recreational cricket player, 180 cm tall, weighing 72 kg, presented with the primary complaint of dull aching pain in the bilateral buttock region. The onset of this condition was sudden, following a traumatic fall experienced by the patient while attending to personal hygiene six months back. He reported experiencing a feeling of fall and subsequently sought support before going to bed. The following morning he encountered a similar fall, and immediately he went to the hospital with a friend. In the orthopedic department, senior residents examined him but failed to establish a definitive diagnosis. An X-ray was suggested, showing a reduction in the joint space at the lumbar region but it was inconclusive; after that further investigation was done through magnetic resonance imaging (MRI). The MRI findings indicated disc bulging at L3-L4, and L4-L5 levels and lumbar sacralization of the L5 vertebra, which aligned with the bilateral radiating pain experienced in his legs. This pain rendered his legs unstable, resulting in frequent buckling and making walking without support difficult. He was confined to complete bed rest for 15 days as advised, following which medications were initiated and he was mandated to undergo physiotherapy. Seeking further assistance, he went to sports physiotherapy, where specialized clinical examinations were done. Figure 1 shows the region of pain in the animated body chart of the patient.

Examination

The patient reported a pain that was dull aching in nature, rated at 7/10 on the Numeric Pain Rating Scale (NPRS) localized in the region just slightly proximal and medial to the greater trochanter, leading to difficulty in independent walking and limitations in activities involving walking and running. During observations, he displayed intact general motor function. Positive point tenderness of grade 2, i.e. patient complains of pain and winces when palpated at the gluteal region just slightly proximal and medial to the greater trochanter. Additionally, muscle spasms were confirmed around the piriformis area. Special tests, particularly the piriformis tightness test, displayed positive results, reinforcing the diagnosis. On the basis of the above assessment, the patient was a suspected case of piriformis syndrome. The protocol for his treatment was initiated following this diagnosis.

Functional Assessment

Range of motion measurement is an ideal measure to asses flexibility and the impact of injury on movement. A goniometer was used to assess the ranges for the lower limb. The flexion range of motion for the Hip Joint was 0-70^o^ on the right side and 0-65^o^ on the left side. Both right and left sides had an extension range of 0-20^o^. The abduction and internal rotation ranges were 0-30^o^ for both right and left sides. Adduction had a range of 0-20^o^ for both right and left sides. The external rotation range was 0-30^o^ for the right side and 0-35^o^ for the left side. As for the knee joint, the flexion range of motion was 0-100^o^ for both the right and left sides. The extension range was 100^o^-40^o^ for the right side and 100^o^-50^o^ for the left side. In terms of the ankle, dorsiflexion had a range of 0-10^o^ for both right and left sides, while plantarflexion had a range of 0-30^o^ for both right and left sides. Muscle strength was assessed as per Oxford gradings of manual muscle testing. The hip flexors, hip extensors, hip external rotators, and hip internal rotators have a 3+, indicating a fair plus performance, i.e. they can complete the range of motion against gravity with minimal resistance. The hip abductors have a 3, i.e. he is able to achieve the range of motion against gravity. The hip adductors, knee flexors, and extensors have a 4, indicating a range of motion against gravity with moderate resistance. Reflexes and sensory assessment were done and it was found that reflexes were intact, there was no sensory impairment, and that all senses were intact. Post evaluation, it was clearly signified that the patient’s strength and ranges both were significantly affected due to pain.

Diagnostic Assessment

**Figure 2:**
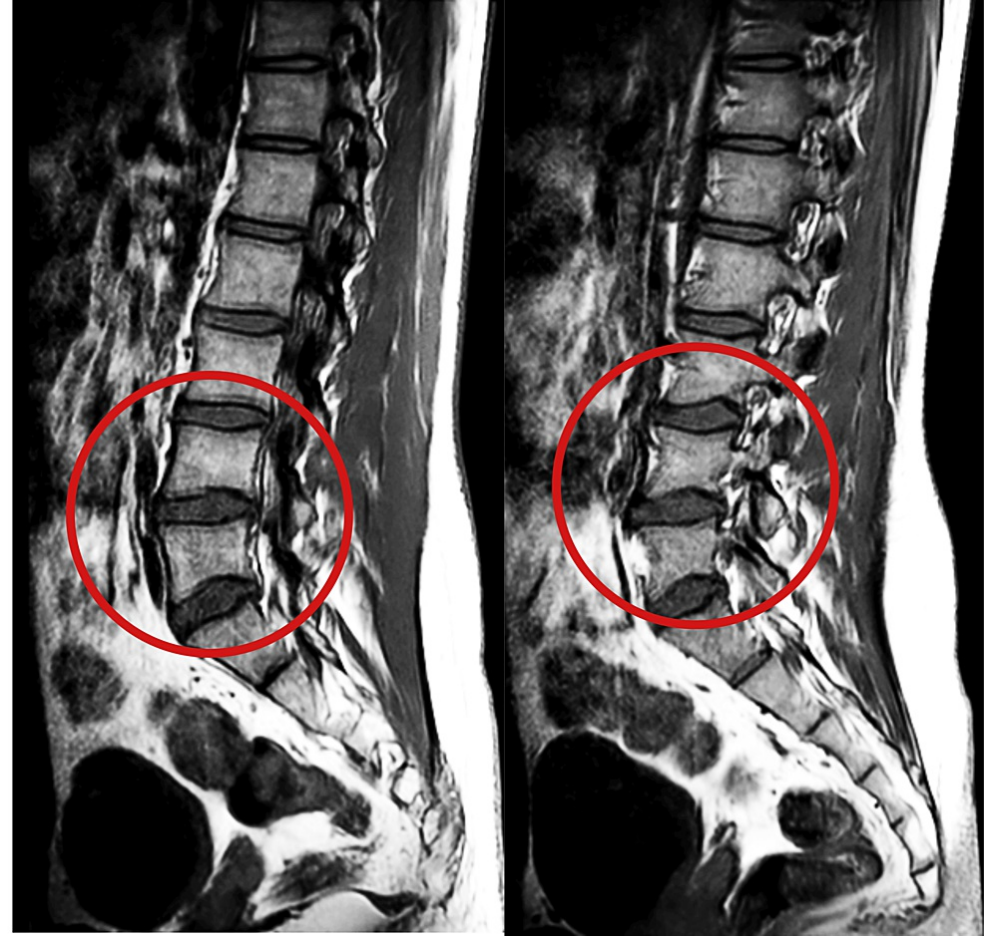
MRI of the lumbar spine. MRI: Magnetic resonance imaging. Bulging disc at the following levels: L3-L4 and L4-L5.

MRI of the lumbar spine dated May 13, 2023, reveals a bulging disc on the following levels: L3-L4, L4-L5, and also lumbar sacralization of the L5 vertebra. Figure 2 shows the MRI of the lumbar spine.

Intervention

A six-week physiotherapeutic treatment program was tailored for the patient 5 times a week for around one session of 45 minutes each day. The treatment goals encompass a comprehensive approach to address various aspects of the patient's condition. Firstly, the focus lies on releasing muscle tightness to ease discomfort. Simultaneously, the aim is to alleviate pain, improve the range of motion, and reduce radiating pain, thereby enhancing overall function and mobility. Strengthening the muscles surrounding the knee, quadriceps, and adductors to ensure greater stability.

Additionally, special attention is directed towards reinforcing the abductor muscles to support gait and stability. Lastly, stability strategies are integral, intending to enhance stability and overall control for improved functional movement and reduced discomfort. These goals collectively form the core objectives of the treatment plan, aiming for holistic improvement and sustained relief for the patient. A detailed summary of therapeutic intervention is mentioned in Table 1.

**Table 1:** Summary of the interventions. MET: Muscle energy technique, NM: Neurodynamic mobilization, ROM: Range of motion.

Structure affected	Goals	Intervention	Rationale
Piriformis muscle	Muscle tightness release	Passive piriformis stretch (30 seconds hold x 3 repetitions on both sides).	The piriformis muscle can become tight and cause discomfort and limited range of motion. Stretching can help alleviate this [13].
Piriformis muscle, hip joint	Pain relief, improvement of ROM	MET for piriformis muscle with hip at 60-degree flexion. Ten repetitions of resistive isometric given to abduction for 7-10 seconds. The initial efforts should be 20 percent of the patient's strength.	The manual therapy known as MET lengthens and relaxes muscles by applying mild isometric contractions. This results in a reduction in piriformis muscle stiffness, an improvement in range of motion, and immediate pain alleviation [14].
Sciatic nerve	Reducing radiating pain	Passive neural tissue mobilization for lower limb bilaterally (10 repetitions) –sliding mobilization of the sciatic nerve is involved in the NM technique. The NM approaches were administered to the affected lower leg three times over the course of a four-minute session, with a one-minute rest in between each application. The proximal slider, which included raising the leg straight and applying plantar flexion without causing any discomfort, and the distal slider, which involved dorsiflexing the foot during the leg's flexion phase, comprised the NM of the sciatic nerve. These movements were alternated at a pace of around two seconds every cycle; one, a second spent in flexion and another in extension.	This technique is aimed at reducing symptoms associated with issues related to the sciatic nerve, such as pain or discomfort in the leg. The goal of this method is to lessen the physical strain on nerves [15].
Hamstrings, quadriceps, gluteal muscles	Strengthening of the surrounding muscles	Isometrics hams quads glutes (10 repetitions x 10 seconds hold x 3 sets).	Isometric exercises can help to strengthen the muscles without moving the joint and can therefore be useful in cases where movement causes pain.
Hip abductors	Abductor strengthening	Clamshells exercise (10 repetitions x 3 sets).	The clamshell exercise targets the hip abductor muscles, which are important for gait and stability.
Quadriceps	Strengthening of quads (for stability of the knee)	Quad chair (10 repetitions x 3 sets), mini squats (10 repetitions × 3 sets).	Strengthening the quadriceps can help to improve knee stability.
	Neuromuscular training strategies	The ankle approach is a single-segment inverted pendulum that is controlled by the torque generated by the ankle joint. It entails a pure rotation of the body around the ankle joint. The hip technique entails twisting the upper body in forward and downward which causes the lower body to rotate backward.	Over 90% of the time, the ankle approach is the most often utilized control strategy for standing balance [14]. The biomechanical processes behind the ankle and hip techniques differ from one another. While the hip technique indirectly regulates the global center of mass by managing the local center of mass of the upper body, the ankle strategy concentrates on controlling the center of pressure with respect to the center of mass [15].

Outcome measures

Following a six-week intervention, the patient's pain score on the NPRS was 2/10 post-treatment. Evaluation of muscle strength and range of motion revealed significant improvement. The hip flexors, extensors, abductors, adductors, and external and internal rotators all achieved a grade of 5, indicating good performance. The knee flexors and extensors also showed a grade of 5, indicating good performance in completing the range of motion against gravity with maximum resistance. This shows overall improvement in strength and positive outcomes of the treatment. For the hip joint, the range of motion for flexion was improved to 0-100^o^ for both right and left. Extension, abduction, adduction, and internal rotation all had an improved range of 0-30^o^ for right and left. The external rotation had an improved range of 0-35^o^ for the right and 0-40^o^ for the left. For the knee joint, the range of motion for flexion had improved to 0-100^o^ for both right and left. An extension had an improved range of 100^o^-80^o^ for both right and left. The ankle dorsiflexion had an improved range of 0-20^o^ for the right and 0-10^o^ for the left. Plantarflexion had an improved range of 0-30^o^ for the right and 0-40^o^ for the left. This overall improvement in the range of motion is a positive outcome of the treatment.

## Discussion

A 19-year-old with PIVD and piriformis syndrome underwent comprehensive physiotherapy rehabilitation, focusing on pain relief, neural tissue tension release, strength improvement, and neuromuscular training. The patient showed significant improvement during follow-up sessions and the exercise program was further progressed.

A study by Kutty RK et al. compared traditional physical therapy with neural mobilization in treating piriformis syndrome. The study included 42 participants aged 30-50, divided into two groups: one receiving neural mobilization and traditional physical therapy and the other receiving conventional physical therapy. The results showed significant differences in VAS (visual analog score) and range of motion hip scores between the control groups and experimental groups. These findings support that treating piriformis syndrome with a combination of neural mobilization and traditional physical therapy is more successful than treating the condition with traditional therapy alone, both in the initial and final stages of treatment [16]. Villafañe JH et al., in their study, included neurodynamic mobilization; soft tissue work on the hamstring and psoas muscles was put into place [15]. The following outcome measures were measured: range of motion, manual muscle testing, pressure pain threshold, and a numerical pain rating scale. They were evaluated prior to therapy, one week after treatment, and three months later. Results revealed that after three sessions over a week, the patient's left foot had recovered to full function with a decrease in subjective discomfort and improvements in strength and range of motion [15]. With this evidence, the technique was integrated with the above protocol.

The intricate relationship between sensory input, cerebral processing, and efferent output is represented by neuromuscular control. Any degree of impairment can change neuromuscular control, which can then cause injury or lower functional levels. Identification of altered neuromuscular control will be aided by an understanding of how injury affects proprioception, dynamic joint stability, postural control, and the sensorimotor system [17]. In healthy young people, the study examines the biomechanical control systems that underlie body sway during unperturbed upright standing. It proposes that the ankle and hip joint oscillations can be explained by a “hybrid control system” that uses one active and one passive component for the ankle and one for the hip joint, respectively. This model is extended to the ankle and hip joints in the research, with separate parameter gains added for each joint. The expanded model can reproduce both tactics, according to simulations, with the pure ankle strategy being more resilient and energy-efficient. Hip and ankle control are used in mixed strategies, which are not advantageous unless control improvements are at the edge of stability and result in a stable mixed strategy. The hip strategy primarily involves delayed activation of trunk and thigh muscles radiating proximal to distal to other muscles while the ankle strategy involves delayed activation of ankle muscles followed by distal-to-proximal thigh and trunk activation [18]. The multifaceted approach addressed not only muscular components but also neural aspects, aiming to restore peripheral nervous system balance and reduce symptoms associated with sciatic nerve compression. Additionally, the emphasis on neuromuscular control underscores the importance of understanding altered neuromuscular patterns in injury and the potential benefits of targeted interventions to improve functional outcomes. The biomechanical control systems identified in healthy individuals provide a theoretical basis for the effectiveness of the ankle and hip strategies employed in the rehabilitation protocol. 

## Conclusions

A comprehensive physical therapy program was administered to a 19-year-old individual suffering from piriformis syndrome and prolapsed intervertebral disc. This holistic approach led to notable enhancements in alleviating pain, relaxing nerve tension, and strength, and improving functional capabilities. The regimen encompassed strategies for pain management, nerve exercises, strength development, and muscle coordination exercises, all contributing to the overall well-being and functional proficiency of the patient. This case report underscores the potency of a well-rounded physical therapy approach in addressing these musculoskeletal conditions, underlining the significance of the given therapeutic interventions.
